# Putrescine Detected in Strains of *Staphylococcus aureus*

**DOI:** 10.3390/pathogens12070881

**Published:** 2023-06-27

**Authors:** Javier Seravalli, Frank Portugal

**Affiliations:** 1 Redox Biology Center and Department of Biochemistry, University of Nebraska-Lincoln, Lincoln, NE 68588, USA; jseravalli1@unl.edu; 2 Department of Biology, The Catholic University of America, Washington, DC 20064, USA

**Keywords:** putrescine, *Staphylococcus aureus*, *N*-acetyl-putrescine, bacterial pathogen, mass spectrometry, multiple reaction monitoring (MRM)

## Abstract

Most forms of life, including the archaea, bacteria, and eukaryotes synthesize the polyamine putrescine. Although putrescine is widely distributed, several Gram-positive bacteria, including *Staphylococcus aureus* (*S. aureus*), appear to be the exceptions. We report here that strains of *S. aureus* can produce the polyamine putrescine, as well as the derivative *N*-acetyl-putrescine. Three strains of *S. aureus* from the American Type Culture Collection (ATCC), one strain listed in the National Center for Biotechnology Information (NCBI) database, whose genomic sequence is well defined, and well as eight strains from *S. aureus*-induced brain abscesses of individual patients from multiple geographic locations were evaluated. Each strain was grown in complete chemically defined medium (CDM) under stringent conditions, after which the partially purified conditioned medium (CM) was analyzed by mass spectroscopy (MS), and the data were reported as the *ratio* of experimental results to controls. We confirmed the synthesis of putrescine by *S. aureus* by using ^13^C/^15^N-labeled arginine as a tracer. We found that agmatine, *N*-acetyl-putrescine, ornithine, citrulline, proline, and NH_3_ were all labeled with heavy isotope derived from ^13^C/^15^N-labeled arginine. None of the strains examined produced spermine or spermidine, but strains from either ATCC or human brain abscesses produced putrescine and/or its derivative *N*-acetyl-putrescine.

## 1. Introduction

**Figure 1:**
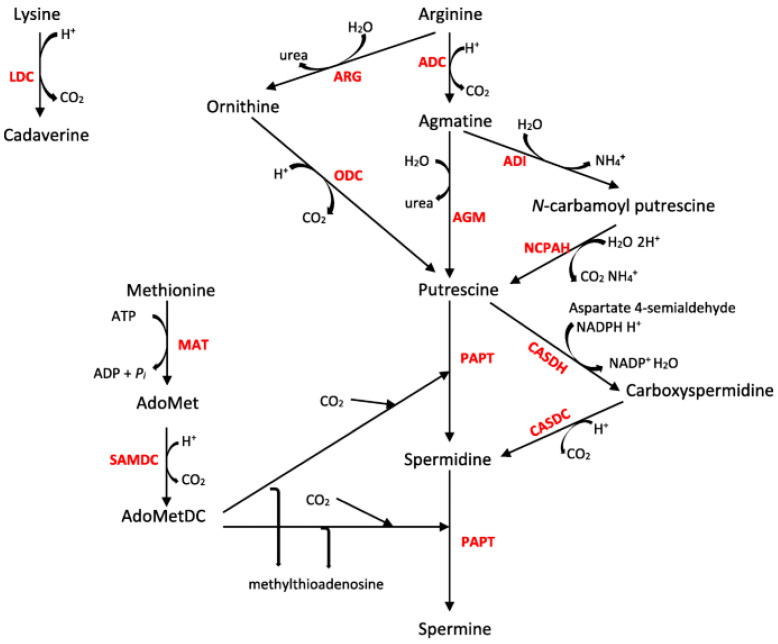
Polyamine biosynthesis in *S. aureus* showing interrelationships of putrescine, arginine and ornithine production [1]. Decarboxylation of ornithine produces putrescine. ARG, arginine; ADC, arginine decarboxylase; ARG, arginase; ODC, ornithine decarboxylase; AGM, agmatinase; ADI, agmatine deiminase; NCPAH, N-carbamoylputrescine amidohydrolase; CASDH, carboxyspermidine dehydrogenase; CASDC, carboxyspermidine decarboxylase; PAPT, polyamine aminopropyltransferase; MAT, methionine adenosyltransferase; SAMDC, S-adenosylmethionine decarboxylase; LDC, lysine decarboxylase; AdoMet, S-adenosylmethionine; AdoMetDC, decarboxylated S-adenosylmethionine.

Despite the ability of archaea, prokaryotes, and eukaryotes to produce polyamines, researchers have held that the human bacterial pathogen *Staphylococcus aureus* (*S. aureus*) appears to be an exception. Figure 1 shows the biochemical pathways in Gram-positive bacteria, in which arginine is converted to putrescine. Arginine can be decarboxylated to agmatine via arginine decarboxylase, which is then enzymatically converted to putrescine by agmatinase. Alternatively, agmatine, via *N*-carbamoyl-putrescine, can be converted to putrescine through the action of agmatine deiminase. Arginine can also be converted to ornithine by arginase, followed by the conversion of ornithine to putrescine by ornithine decarboxylase. However, as strains of *S. aureus* lack these reported enzymes, other than arginase and arginine decarboxylase, the question of how ornithine or agmatine are converted to the final product of putrescine in *S. aureus* remains unclear.

The idea that *S. aureus* cannot carry out the biosynthesis of putrescine is based on three lines of evidence: first, on the reported lack in the genome of *Staphylococcus epidermis*, a species related to *S. aureus*, of an ornithine decarboxylase; agmatinase; agmatine deiminase; or *N*-carbamoylputrescine amidohydrolase gene needed for the synthesis of the polyamine putrescine [2]; second, on the ability of the polyamines spermine and spermidine to inhibit the growth of *S. aureus*, thereby making the synthesis of these two polyamines unlikely [3]; and, third, on the inability of cultures of *S. aureus* to produce putrescine when chemically defined medium (CDM) free of polyamines is used for growth [4].

However, despite these reports, the possibility that *S. aureus* can synthesize putrescine, particularly in view of the fact that it appears to be an exception, warrants reconsideration. First, the genome of *S. aureus* does encode the putative polyamine biosynthetic enzyme SACOL0523, an ornithine/arginine/lysine decarboxylase. This enzyme can catalyze the formation of putrescine, agmatine, or cadaverine, depending on substrate specificity, and supports the possible synthesis of putrescine in *S. aureus* [3]. Second, however, the absence of homologues to SAM-decarboxylase or spermidine synthetase in the genomes of 13 *S. aureus* species confirms that *S. aureus* cannot synthesize spermidine from putrescine by the canonical pathway [3].

Whether *S. aureus* produces putrescine is essential not just to understanding the metabolism of this organism but to extending knowledge of the basis for its severe pathogenicity. For example, putrescine can reduce antibiotic-induced oxidative stress, thereby enhancing antibiotic resistance [5]. Pathogenic bacteria such as *S. aureus* potentially use polyamines to modulate gene expression during infection [6].

Therefore, we tested the hypothesis that *S. aureus* could synthesize putrescine under the appropriate growth conditions. We did so by growing 12 strains of *S. aureus* in CDM containing FeSO_4_ [7]. The results indicated that 33% of the strains tested here, whether genome documented or patient derived, produced either putrescine or its derivative *N*-acetyl-putrescine when either un-labeled or ^13^C/^15^N-labeled arginine was used as the precursor. In addition, the concentration of putrescine produced by these cultures was comparable to that found in eukaryotic cells.

## 2. Materials and Methods

Chemicals of analytical grade were purchased from Sigma-Aldrich (Saint Louis, MO, USA), with the exception of glucose and glycine (Honeywell Fluka, Morris Plains, NJ, USA), hydrochloric acid (J.T. Baker, Phillipsburg, NJ, USA), and sodium chloride (Acros Organics, Newark, NJ, USA). Trypticase soy broth (TSB) was obtained from Becton, Dickinson and Company (Franklin Lakes, NJ, USA). Chemicals used for mass spectrometry (MS) analysis included glacial acetic acid (Sigma-Millipore, St. Louis, MO, USA); ammonium hydroxide (Thermo Fisher Scientific, Washington, DC, USA), used to adjust the pH to 9.50; and acetonitrile (Optima LC-Grade, Thermo Fisher Scientific, Washington, DC, USA).

*S. aureus* strains BAA-44, ATCC 43300, and ATCC 25293 were obtained from the American Type Culture Collection (Manassas, VA, USA). Seven strains from *S. aureus*-induced brain abscesses of individual patients were provided by David Lonsway of the Centers for Disease Control and Prevention (CDC, Atlanta, GA, USA), which we numbered AR1454, AR2789, AR3175, AR42208, AR 5738, AR6498, and AR7067. Kristina Hulten at Baylor College of Medicine (BCM, Houston, TX, USA) shared a MRSA USA300 strain from a brain abscess of a pediatric patient. Alexander Tomasz at The Rockefeller University (NY, NY) provided Col-S, derived from the parental MRSA strain COL, from which the resistance staphylococcal cassette chromosome *mec* was excised. This strain and the three ATCC strains had fully sequenced and annotated genomes. Contributors of the strains also provided the minimum inhibitory concentration (MIC) data for antibiotic resistances of the strains.

The hemolytic activity of each strain was measured by growth on triplicate TSB agar plates containing sheep blood (Hardy Diagnostics, Albany, NY, USA) for 24 h at 37 °C. Hemolysis was determined by visual analysis with a New Brunswick Scientific plate reader (Enfield, CT, USA). The concentrations of viable cells in CDM were determined by growth on triplicate plates of TSB agar. The colony-forming units (CFU) were measured with a New Brunswick Scientific plate reader. The total cell concentrations were determined with a Beckman DU spectrophotometer at 600 nm.

Each strain of *S. aureus* was grown in TSB twice, and cultures were stored in 1.5 mL plastic centrifuge tubes, each containing 850 µL of TSB and 150 µL of 80% glycerin (Thermo Fisher Scientific, Washington, DC, USA) at −80 °C to maintain viability. The composition of the CDM (per 100 mL) included 100 mg KH_2_PO_4_, 120 mg alanine, 36 mg arginine, 12 mg cysteine-HCl, 120 mg glycine, 120 mg glutamic acid, 24 mg histidine-HCl. H_2_O, 30 mg isoleucine, 30 mg leucine, 30 mg lysine-HCl, 9 mg methionine, 10 mg phenylalanine, 120 mg proline, 240 mg serine, 240 mg threonine, 3 mg tryptophan, 10 mg tyrosine, 24 mg valine, 120 mg aspartic acid, calcium pantothenate 50 µg, biotin 0.3 µg, thiamine-HCl 50 µg, niacin, 50 µg, NaCl 400 mg, NH_4_Cl 50 mg, Na_2_HPO_4_ 175 mg, MgSO_4_ 34.2 mg, glucose 1000 mg, and FeSO_4_·7H_2_O 1 mg.

Each experiment used 100 mL of CDM distributed as 6.25 mL of CDM in each of 16 25 mL Erlenmeyer flasks, into which 50 µL of each *S. aureus* strain was added. Each strain of *S. aureus* or control experiment was grown in CDM using a G76D gyrorotatory water bath shaker (Edison, NJ, USA) at 37 °C and 60 rpm for 24 h. The conditioned medium (CM) was centrifuged at 6000 rpm for 15 min at 4 °C in a Sorvall Rc5C Plus centrifuge using an HS-4 swinging bucket rotor to remove cells. The decanted media was stored at −80 °C until further use. For each experiment, two flasks with only medium were used as controls to confirm the maintenance of sterility. Cultures were prepared in a Nuare Class II Type B/A3 (Plymouth, MN, USA) biological hood.

For purification of the metabolites, a 100 × 1.5 cm column filled with IonSep CM52-cellulose cation exchanger (Biophoretics, Sparks, NV, USA) was used at a temperature of 25 °C. The CM-52 was prepared and washed first with 2 M NaCl, 0.01 M NH_4_HCO_3_, pH 7 buffer. The column was equilibrated with 0.01 M NH_4_HCO_3_, pH 7 buffer. The column was run at a flow rate of 0.6 mL/min and a temperature of 25 °C. Each CM sample, from which cells had been removed, was diluted twofold, with an equal volume of 0.01 M NH_4_HCO_3_, pH 7 buffer, titrated with HCl to pH 4.0 and passed over the prepared column. After washing the column with 0.01 M NH_4_HCO_3_, pH 7 buffer to remove unbound material, bound metabolites were eluted with 0.7 M NH_4_HCO_3_. Eluant was collected in 1 mL portions in glass tubes and read at both A260 and A280 nm. The results were graphed in Microsoft Excel, and the resulting profile was compared with a standardized profile. Tubes containing the leading peak were pooled. This portion of the eluant was reduced to dryness by lyophilization, re-dissolved in a minimal amount of Millipore purified water, re-lyophilized, heated overnight at 60 °C in a convection oven, and then tested for the absence of NH_3_ with Hydrion Ammonia Test paper (Micro Essential Laboratory, Brooklyn, NY, USA). Media controls consisted of the exact same steps described above, with the exception of no addition of strains of *S. aureus* to the CDM. Analyzed by MS, the resulting data for the experiments relative to the controls represented the average of 3 independent experiments and 3 independent controls for each, together with the standard deviation for the ratios. These ratios may have represented the lower limit of the metabolomic changes that could have been occurring in these cultures if a component in the CDM was metabolized by *S. aureus*.

The LC-MS/MS analysis was performed on a HPLC LC-1200 system (Agilent, Santa Clara, CA, USA), interfaced to 4000-QTrap (Sciex, Framingham, MA, USA), operating in Multiple Reaction monitoring (MRM) mode with a dwell time of 10 ms per transition. The sample pellets were suspended in 1000 μL of LC-grade water and injected onto a 4.6 × 100 mm Amide X-Bridge (2.5 um, Waters, Milford, MA, USA) running at 0.5 mL/min. The gradient consisted of 20 mM ammonium acetate, 20 mM ammonium hydroxide pH 9.5 (A), and LC-grade acetonitrile (B). The gradient was as follows: 2 min hold after injection at 95% B, followed by a 20 min linear gradient from 95% to 5% B, then by a 5 min hold at 5% B, and, finally, a re-equilibration for 15 min at 95% B. The ESI Ion-Source was operated at 500 °C, source gasses 1 and 2 at 70 L/min, and electrospray potential at 5000 V and −4200 V in positive and negative modes, respectively. MRM data were collected in positive and negative ion modes with separate injections of 5 μL and 15 μL, respectively. The MRM chromatograms using MetaboAnalyst 3.0 and Microsoft Excel was used for statistical analysis, with Sigmaplot 14.0 for chromatogram plotting. The time point samples were analyzed as biological triplicates. The chromatogram areas were normalized against the area for ^15^N_1_–^13^C_5_-labeled proline added as an internal standard (2.31 μM) for the experiments using unlabeled arginine. Compounds identified in the negative ionization mode method were normalized with the total ion chromatogram, and these data presented as % area gram^−1^.

**Figure 2:**
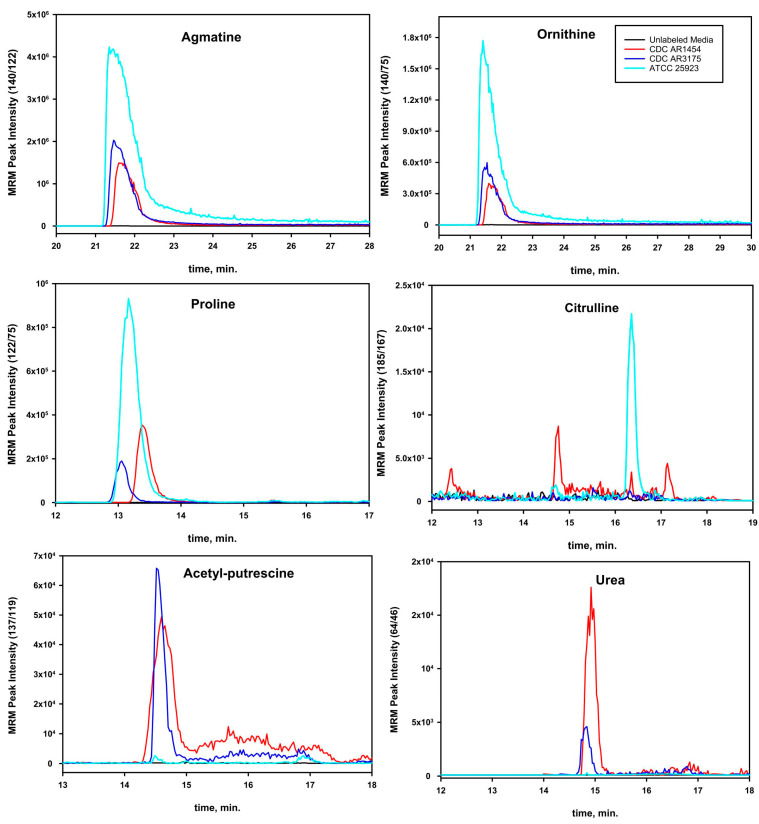
LC-MRM chromatograms of the incorporation of ^13^C and ^15^N into agmatine, ornithine, proline, citrulline, *N*-acetyl-putrescine, and urea from ^13^C^15^N-arginine.

Further MS experiments were conducted with ^13^C/^15^N-labeled arginine-HCl (Thermo Fisher Scientific, Washington, DC, USA) in order to determine the possible origin of putrescine metabolites found in the CM. Samples in the lyophilized state were suspended into 0.5 or 1.0 mL of LC-grade water (Thermo Fisher Scientific, Washington, DC, USA). The MRM transitions for the labeled metabolites (Q1/Q3) are indicated in the *y*-axis of the chromatograms shown in Figure 2. The MRM transitions were modified to account for the isotopic enrichment, based on knowledge of the mechanism of fragmentation and assuming the same instrument parameters (collision energy, de-clustering potentials, entrance/exit voltages) for the labeled metabolites as for unlabeled metabolites. The parameters for all the MRM runs were as follows: temperature, 550 °C; electrospray potential, +5000 V; curtain gas, 20 L/min; ion-source gas 1, 70 L/min; ion-source gas 2, 70 L/min; collision energy range, 10–30 V; collision cell entrance potential, 10 V; collision cell exit potential, 15 V; and de-clustering potential, +80 V. The total cycle time for the monitored transitions was ~1 s.

## 3. Results

The CDM composition used in this study more closely resembled secretions of the human body than the TSB medium does. Conditions of low oxygen tension (pO_2_) similar to those likely encountered by bacteria when infecting human tissues were also used [8]. This particular adjustment may have been important because *S. aureus* has been classified as a facultative anaerobe. Oxygen and carbon dioxide concentrations have been shown to regulate toxic shock syndrome, alter expression of other virulence factors, and affect quorum sensing [9,10].

Wang reported that soy meal contains the polyamines putrescine, spermine, and spermidine [11]. As 10% of TSB consists of a papain digest of soy meal, it was necessary to test for the possible carryover of TSB present in stocks of each strain when stored at −80 °C. We conducted several control experiments to rule out this possibility. First, in triplicate experiments, CDM medium was incubated with BAA-44 cells that had been stored in 85% TSB. The cells were grown for 24 h under our experimental conditions, and the media was then purified and analyzed by MS. These results were compared with triplicate experiments that used CDM without cells that was incubated and analyzed in identical fashion. No significant differences between the BAA-44 experimental samples and CDM controls were detected for putrescine, *N*-acetyl-putrescine, spermidine, or spermine.

Next, BAA-44 cells stored in 85% TSB were grown in triplicate experiments in CDM, to which 4% (*v*/*v*) TSB was added. The CM was then purified, as described in the methods section, and analyzed by MS. The results were compared with triplicate controls of CDM alone, treated, and analyzed in an identical manner. No significant levels of putrescine, *N*-acetyl-putrescine, spermine, or spermidine were found in the CDM/4% TSB samples. Finally, BAA-44 cells stored in 85% TSB were grown in 100% TSB, and the experiments, purifications, and MS analyses were repeated in an identical fashion. No significant amounts of polyamines, with the exception of *N*-acetyl-putrescine (2.42 ± 1.175 at 95% confidence level), were detected in triplicate TSB experimental samples compared to triplicate controls.

For each of the 12 strains in this study, the contents of the polyamines, putrescine, spermine, and spermidine were measured in CM and normalized to each potential polyamine in CDM. By presenting the ratio of experimental data to controls, only the results significantly (90–99% confidence level) greater than one were used to determine the presence or absence of any particular polyamine being produced by *S. aureus* cells. The results in Table 1 show that of the 12 strains of *S. aureus* tested, four either produced statistically significant amounts of putrescine, *N*-acetyl-putrescine, or both. The results suggested that the increased content of putrescine or *N*-acetyl-putrescine in the media came from the production of the metabolites by the cells themselves.

However, statistically significant levels of spermine or spermidine were not found in any of the ratios calculated for experimental results to controls for the 12 strains tested. as might be expected if there were carryover of TSB from the frozen sticks of cells. This suggested that the concentration of TSB used for storage of the bacterial strains was unlikely able to account for the polyamine putrescine found in the medium conditioned by the growth of *S. aureus*.

Genomic identification of ATCC strains BAA-44 and 25923 showed that they each contained the gene for arginine decarboxylase. The action of this enzyme on the amino acid arginine produced agmatine, a putrescine precursor. In addition, both strains contained multiple *N*-acetyl-transferases. The presence of genes for both enzymes suggested that each strain had the potential to produce agmatine, a precursor of putrescine, *N*-acetyl-putrescine, or both. Whether *S. aureus* could convert agmatine to putrescine, and whether *S. aureus* could convert putrescine to *N*-acetyl-putrescine, was a subsequent focus of study.

To determine whether *S. aureus* could synthesize putrescine and/or its derivative *N*-acetyl-putrescine, fully ^13^C/^15^N-labeled arginine was used as a tracer. The results shown in Figure 2 indicate that ^13^C/^15^N-labeled agmatine, ornithine, citrulline, urea, proline, and *N*-acetyl-putrescine were found for the strains CDC AR1454, CDC 3175, and ATCC 25923. Isotope reagent limitation did not allow the same test to be carried out for the other strains of *S. aureus* used in this study. The incorporation of ^13^C/^15^N into agmatine and the other listed metabolites was consistent with the expected metabolic pathways (Figure 1). Moreover, the lack of ^13^C incorporation observed in the acetyl carbons of acetyl-putrescine was consistent with putrescine biosynthesis from ornithine or agmatine; the carbons were not enriched because they originated in the general metabolic pool.

The possibility that a processing step in these experiments could have led to a *chemical* rather than an enzymatic conversion of ornithine to putrescine was further considered. The evidence from *Arabidopsis* showed that extracts from the plant non-enzymatically decarboxylated ornithine to putrescine despite the absence of ornithine decarboxylase [12]. In addition, Wong et al. noted that ornithine could be chemically decarboxylated to putrescine when heated in aqueous solution in the absence of oxygen [13]. To test for chemical decarboxylation while processing CM for MS analyses, the following test was conducted: 25 mL of 0.7 M NH_4_HCO_3_ was added to 5mM ornithine monohydrochloride. The sample was lyophilized twice, as described in the methods section, and then the sample was heated overnight in a convection oven at 60 °C. This sample together with a sample of the untreated ornithine monohydrochloride was analyzed by MS. No evidence of putrescine was found in either the treated or untreated ornithine monohydrochloride (data not shown) sample.

Finally, each strain from ATCC, CDC, and BCM was checked for antibiotic resistance, hemolytic activity, and colony-forming units (CFU) to see whether a relationship with putrescine or *N*-acetyl-putrescine production might exist. These data together are shown in Table 2. The results for the CFU following a 24 h incubation in the CDM ranged from 1.3 to 4.3 × 10^9^/mL. We found no evident correlations between antibiotic resistance, hemolytic activity, or CFU per mL of culture for the strains that produced putrescine and/or *N*-acetyl-putrescine (ATCC 25923, CDC AR1454, CDC AR3175) when either unlabeled arginine or ^13^C/^15^N-labeled arginine was used.

To search for candidate proteins that might have been responsible for the synthesis of putrescine in *S. aureus*, the five *N*-terminal amino acids as a single unit for each protein encoded in the genome of ATCC25923, found to produce putrescine, were used to search the entire proteome of ATCC43300, a strain found not to produce putrescine (Table 3). More than 90 possible candidates were found that were hypothetical proteins whose sequences, but not functions, were known. Deciphering which of these proteins might be involved in the biosynthesis of putrescine in *S. aureus* was beyond the scope of the present paper.

Table 4 examines a few selected strains of four Gram-positive bacterial species, including *Streptococcus pyogenes*, *Bacillus subtilis*, *S. aureus*, and *Mycobacterium tuberculosis*, as well as two Gram-negative strains, including *Pseudomonas aeruginosa* and *Escherichia coli*. All strains were checked both for proteins to transport putrescine or polyamines and the presence of protein for the biosynthesis of putrescine from arginine by one of three canonical pathways. The strains PS003, HKU419 and TJ11-001 (*S. pyogenes*), ms-2 (*B. subtilis*), and 2014C-3655 and 2014-3347 (*E. coli*) all lacked a transport system for putrescine and also lacked a canonical pathway for the synthesis of putrescine. In contrast, a number of strains in Table 4 did have a transport system for putrescine but lacked evidence of a canonical pathway for its biosynthesis.

The strains of *S. aureus* from ATCC, in particular, showed a specific protein for the export of putrescine not seen in any of the other limited number of strains from the other species examined. The *S. aureus* strains ATCC25923 and ATCC43300, for example, both contain the export protein for putrescine, but ATCC25923 synthesizes putrescine from arginine, whereas ATCC43300 does not. These limited data suggest that the biosynthesis of putrescine also requires a transport system for it, although the converse does not hold. In the pathogenic form of avian *E. coli*, both the transport as well as the biosynthesis of putrescine were essential for growth [14].

## 4. Discussion

The metabolic profiles of *S. aureus* strains have not previously shown the presence of putrescine [15,16,17]. Ammons et al. suggested the presence of putrescine in *S. aureus* cultures, but the authors did not report a correction for the polyamine content of the rich medium used [17]. In the present study, the absence of both spermidine and spermine from cultures of all 12 strains was consistent with the fact that spermine and spermidine are bactericidal for *S. aureus* at physiological concentrations [3]. The absence of these two polyamines further supported the suggestion that there was no observable carryover of putrescine from the minute amount of TSB (6.8 × 10^−3^ µL *v*/*v*) present in each 6.25 mL incubation of culture.

One variable may have been differences in the strains of *S. aureus* used in the published studies compared with those used in the present study. Another variable might have resulted from the incubation conditions used here, including the cultures in small 25 mL flasks incubated under relatively low revolutions that may have produced conditions such as low oxygen tension, similar to those encountered by *S. aureus* in the body.

How *S. aureus* produces putrescine is undetermined. Genomic analyses of *S. aureus* strains from the ATCC collection, including those used in this study such as BAA-44, ATCC 25923, ATCC 43300, and COL, as well as the additional strains ATCC 6538, ATCC 9144, ATCC 12600, ATCC BAA-1680, and ATCC 27660, all show the presence of arginine decarboxylase but the absence of agmatine ureohydrolase or agmatinase and *N*-carbamoylputrescine amidohydrolase. In contrast, all of these strains contain ornithine carbamoyltransferase and ornithine aminotransferase.

Putrescine may be produced by the decarboxylation of ornithine under the appropriate culture conditions. Alternatively, it is possible that *S. aureus* may have a previously undisclosed pathway for the synthesis of putrescine, which will be the focus of a separate study. More than one-third of the genes in *S. aureus* code for hypothetical proteins, whose functions remain unknown. ATCC 25923, for example, has 928/2576 genes listed as producing hypothetical proteins (36%). Other *S. aureus* strains show similar properties: ATCC BAA-44 reports 1063/2795 (38%), ATCC 43300 has 981/2714 (36%), ATCC 6538 has 905/2572 (38%), ATCC9144 shows 955/2635 (36%), ATCC 12600 lists 868/2529 (34%), ATCC BAA-1680 counts 1046/2754 (38%), and ATCC 27660 finds 963/2647 (36%). A third possibility is the condensation of CO_2_ with an appropriate substrate to produce putrescine, as has been reported for *Chlamydomonas reinhardtii* [18]. However, to see that effect, the experimental conditions required CO_2_-enriched air (3–5% *v*/*v*).

Putrescine is seen in the media of some strains when unlabeled arginine is used but is not seen when ^13^C/^15^N-labeled arginine is used as the initial source. Without yet knowing how *S. aureus* synthesizes putrescine, we can only speculate on explanations for this. When unlabeled arginine was used in growth experiments, 100 mL of CM was applied to the 100 cm chromatography column. When ^13^C/^15^N-labeled arginine was used, only 25 mL of CM was applied to the same sized column. Slight variations in the chromatographic profiles when just 25 mL of CM was used might have possibly accounted for the different results seen for the unlabeled arginine experiments compared to the experiments that incorporated ^13^C/^15^N-labeled arginine into the cells. Neither putrescine nor *N*-acetyl-putrescine (nor any of the other metabolites shown in Figure 2) were detected by LC-MS/MS in control media, in which the cells had not been grown, but which contained either ^13^C/^15^N-labeled or unlabeled arginine following the chromatography procedures.

Better understood is the conversion by *S. aureus* of putrescine to *N*-acetyl-putrescine. Putrescine is acetylated by one or more *N*-acetyl-transferases. Acetylation of putrescine may occur in the medium, possibly due to the release of an enzyme liberated by lysed cells [19].

An important question yet to be answered is whether putrescine in the medium from *S. aureus* serves a useful function for pathogenicity. While the most common diamine present in bacteria generally is putrescine, there is no known conserved function of any polyamine in bacteria [20]. The amount of putrescine produced by some *S. aureus* strains in this report may, *by calculation*, be sufficient to affect a limited number of eukaryotic cells. The volume of a eukaryotic cell is estimated to be 3.4 × 10^−9^ mL [21]. The putrescine concentration in a eukaryotic pTr2 cell, previously established from elongated porcine blastocysts, has a putrescine concentration of 1.22 µmol/mL [22]. Therefore, a single pTr2 cell contains ~4.15 × 10^−3^ pmol. The amount of putrescine produced by 10^7^ cells of *S. aureus* in this study averaged 3.45 pmol of putrescine produced per mL of media (Table 1). Mice injected with 10^7^ colony-forming units (CFU) of *S. aureus* produced similar bacterial loads in individual organs within 3 days [23]. This indicated that the amount of putrescine produced by *S. aureus* was approximately equivalent to the endogenous amount found in about 750 eukaryotic cells.

Previous studies have reported that putrescine could function as a communication signal for other pathogens [24]. Human eukaryotic cells have the transporter hOCT2 for uptake of exogenous putrescine. Once inside the cell, putrescine, along with spermidine and spermine, is involved in regulating cell proliferation [25,26]. Depending on the exogenous levels, putrescine in eukaryotic cells can stimulate the mTOR signaling pathway, inhibit formation of a modified eukaryotic initiation factor, and/or induce apoptosis, as well as be essential for normal eukaryotic cell viability [22,27].

The cultivation of *S. aureus* under more physiological-like conditions, the particular strains used, the composition of the CDM, and the analyses of the results relative to the medium used here are the variables that may each affect the metabolic profiles of the media. However, given these variables, the evidence obtained here shows that *S. aureus* does produce putrescine and/or *N*-acetyl-putrescine, depending on the strain and the conditions of growth.

## Figures and Tables

**Table 1:** The putrescine concentrations for each strain represent the ratios of putrescine or *N*-acetyl-putrescine in the media from cells grown in CDM to putrescine or *N*-acetyl-putrescine in CDM alone, as shown in columns two and four, respectively. Concentrations of putrescine (per mL of culture, columns three and five) were obtained by interpolation of the concentrations from external calibration curves. Results are at a confidence level of 90% unless otherwise indicated.

Strain	Putrescine	Putrescine (nM)	*N*-Acetyl-Putrescine	Acetyl Putrescine (nM)
ATCC 25923	4.17 ± 3.03	0.4	17.2 ± 15.14	1.65
CDC AR1454	2.66 ± 0.91 ^b^	6	12.59 ± 11.09 ^b^	28.4
CDC AR3175	1.78 ± 0.58	3.95	---	
CDC AR7067	---	---	149.98 ± 123.37 ^a^	14.4

^a^ Confidence level 95%. ^b^ Confidence level 99%.

**Table 2:** Antibiotic resistances, hemolytic activities, and concentrations of *SA* strains used in this study.

Strain	Antibiotic Resistance	Hemolytic Activity (24 h)	Concentration CFU/mL
ATCC BAA-44	Multiple ^1^	None	3.69 × 10^9^
NCBI COL-S	None	None	1.7 × 10^9^
ATCC 43300	Methicillin, Oxacillin	None	2 × 10^9^
ATCC 25923	Cefoxitin, Penicillin, Mupirocin	None	1.8 × 10^9^
CDC AR1454	Oxacillin, Penicillin, Erythromycin	None	4.35 × 10^9^
CDC AR2789	Oxacillin, Penicillin, Clindamycin, Erythromycin, Levofloxacin	None	2.9 × 10^9^
CDC AR3175	Oxacillin, Penicillin, Clindamycin, Erythromycin	None	2.87 × 10^9^
CDC AR42208	Oxacillin, Penicillin, Erythromycin, Levofloxacin	Some	1.3 × 10^9^
CDC AR 5738	Oxacillin, Penicillin, Erythromycin	Some	2.21 × 10^9^
CDC AR6498	Oxacillin, Penicillin, Clindamycin, Erythromycin, Levofloxacin	None	1.9 × 10^9^
CDC AR7067	Oxacillin, Penicillin, Erythromycin, Levofloxacin	Some	2.23 × 10^9^
Baylor MRSA USA300	Erythromycin, Vancomycin, Methicillin	Strong	2.7 × 10^9^

^1^ Ampicillin, amoxicillin/clavulanic acid, ciprofloxacin, cephalothin, doxycycline, gentamicin, erythromycin, imipenem, methicillin, penicillin, tetracycline, oxacillin, azithromycin, clindamycin, ceftriaxone, rifampin, amikacin, streptomycin, and tobramycin. Intermediate resistance to minocycline.

**Table 3:** The first five amino acid residues as one unit at the *N*-terminus of each protein encoded by the genome of ATCC25923, a strain found to produce putrescine (column 2), was used to search for a matching sequence in proteins encoded by the genome of ATCC43300, a strain that was found not to produce putrescine (column 3). The numbers for each hypothetical protein represent the position of the encoded sequence for that protein in the genome.

GENE	25923	43300
Hypothetical protein 132469..132870	1	0
Hypothetical protein 135487..136044	1	2
Hypothetical protein 156985..157179	2	0
Hypothetical protein 157404..158372	2	0
Hypothetical protein 158649..159218	3	2
Hypothetical protein 159559..160086	4	3
Hypothetical protein 160137..160355	2	1
Hypothetical protein 160356..160952	3	2
Hypothetical protein 160964..161305	1	0
Hypothetical protein 161652..162293	3	2
Hypothetical protein 162290..162574	1	2
Hypothetical protein 162576..162938	2	3
Hypothetical protein 164721..165590	1	0
Hypothetical protein 165974..166183	2	0
Hypothetical protein 166176..166322	1	0
Hypothetical protein 166650..167291	1	0
Hypothetical protein 167295..167507	3	2
Hypothetical protein 167661..168323	1	0
Hypothetical protein 177188..177328	3	4
Hypothetical protein 434789..435115	1	2
Hypothetical protein 460317..460508	1	0
Hypothetical protein 490900..491820	1	2
Hypothetical protein 513477..515150	2	3
Hypothetical protein 637833..638594	1	2
Hypothetical protein 689131..690099	2	1
Hypothetical protein 690618..691028	1	3
Hypothetical protein 734010..734387	2	6
Hypothetical protein 734404..735225	2	3
Hypothetical protein 945446..945742	1	0
Hypothetical protein 992307..993047	1	2
Hypothetical protein 1004508..1005284	3	2
Hypothetical protein 1182854..1183327	2	1
Hypothetical protein 1183342..1183716	2	1
Hypothetical protein 1183734..1184123	2	1
Hypothetical protein 1184125..1184517	1	0
Hypothetical protein 1750363..1750452	5	4
Hypothetical protein 1254171..1255004	1	0
Hypothetical protein 1256675..1257370	1	0
Hypothetical protein 1257514..1257726	3	2
Hypothetical protein 1257727..1257999	1	0
Hypothetical protein 1258150..1258359	2	0
Hypothetical protein 1258750..1259619	2	3
Hypothetical protein 1259633..1261342	1	0
Hypothetical protein 1262031..1262672	3	2
Hypothetical protein 1263189..1263530	2	3
Hypothetical protein 1263542..1264138	3	2
Hypothetical protein 1264139..1264357	2	1
Hypothetical protein 1264408..1264935	4	3
Hypothetical protein 1265276..1265845	3	2
Hypothetical protein 1266122..1267090	2	0
Hypothetical protein 1267386..1267667	1	0
Hypothetical protein 1267785..1268297	1	0
Hypothetical protein 1274829..1275455	1	2
Hypothetical protein 1488328..1488564	2	1
Hypothetical protein 1521968..1522474	3	4
Hypothetical protein 1587960..1588349	4	6
Hypothetical protein 1653173..1654051	4	3
Hypothetical protein 1744950..1745777	2	1
Hypothetical protein 1850234..1850428	1	0
Hypothetical protein 1975041..1976078	4	6
Hypothetical protein 2009292..2009777	1	0
Hypothetical protein 2020584..2021309	3	2
Hypothetical protein 2021419..2022144	2	1
Hypothetical protein 2187375..2187581	2	3
Hypothetical protein 2197213..2197338	1	0
Hypothetical protein 2268153..2268722	1	0
Hypothetical protein 2292291..2292692	1	2
Hypothetical protein 2314681..2314902	2	3
Hypothetical protein 2321684..2322388	6	5
Hypothetical protein 2413521..2414975	2	1
Hypothetical protein 2414986..2415288	2	1
Hypothetical protein 2415414..2415788	1	0
Hypothetical protein 2420121..2421611	2	1
Hypothetical protein 2421611..2426260	1	0
Hypothetical protein 2426498..2426944	1	0
Hypothetical protein 2427009..2427962	5	3
Hypothetical protein 2427963..2428343	2	1
Hypothetical protein 2428340..2428717	1	0
Hypothetical protein 2428717..2429052	1	0
Hypothetical protein 2429039..2429371	1	0
Hypothetical protein 2429380..2429538	2	1
Hypothetical protein 2429574..2430821	2	1
Hypothetical protein 2430909..2431493	1	0
Hypothetical protein 2431486..2432736	1	0
Hypothetical protein 2432742..2432981	1	0
Hypothetical protein 2432956..2434650	1	0
Hypothetical protein 2434653..2435120	2	1
Hypothetical protein 2435250..2435603	1	0
Hypothetical protein 2435610..2436062	1	0
Hypothetical protein 2436177..2436611	1	0
Hypothetical protein 2437643..2437795	2	1
Hypothetical protein 2437795..2437995	1	0
Hypothetical protein 2438155..2438400	1	0
Hypothetical protein 2439228..2439476	2	1
Hypothetical protein 2439477..2439836	1	0
Hypothetical protein 2439837..2440022	1	0
Hypothetical protein 2440027..2440431	1	0
Hypothetical protein 2440441..2440662	1	0
Hypothetical protein 2440675..2440833	2	1
Hypothetical protein 2440827..2441606	1	0
Hypothetical protein 2441616..2442386	1	0
Hypothetical protein 2442452..2442733	1	0
Hypothetical protein 2442872..2443543	1	0
Hypothetical protein 2444140..2444919	1	0
Hypothetical protein 2444912..2445133	1	0
Hypothetical protein 2445143..2445403	1	0
Hypothetical protein 2445814..2445975	1	0
Hypothetical protein 2446877..2447104	1	0
Hypothetical protein 2447106..2447297	1	0
Hypothetical protein 2447354..2447893	1	0
Hypothetical protein 2450801..2450926	1	0

**Table 4:** Putrescine production and transport systems in four Gram-positive and two Gram-negative strains.

Strain	Transport	Ornithine Decarboxylase	Agmatinase	Agmatine Deiminase	*N*-Carbamoyl- Putrescine Amidohydrolase	Arginine Decarboxylase
*Streptococcus pyogenes*						
NCTC12064	Spermidine/putrescine transport permease	No	No	No	No	No
HKU488	Spermidine/putrescine transport permease	No	No	No	No	No
MGAS29326	Spermidine/putrescine transport permease	No	No	No	No	No
TJ11-001	No transport	No	No	No	No	No
BSAC_bs1388	ABC transporter permease	No	No	No	No	No
ABC221	ABC transporter permease	No	No	No	No	No
37-97S	ABC transporter permease	No	No	No	No	No
NCTC8324	Spermidine/putrescine transport permease	No	No	No	No	No
HKU419	No transport	No	No	No	No	No
MGAS10786	ABC transporter permease	No	No	No	No	No
21SPY7071	ABC transporter permease	No	No	No	No	No
PS003	No transport	No	No	No	No	No
*Pseudomonas aeruginosa*						
Pa58	Spermidine/putrescine ABC transporter	Yes	Yes	No	Yes	Yes
AR_0353	Polyamine antiporter	No	Yes	Yes	Yes	Yes
AG1	Putrescine ABC transporter	No	No	Yes	Yes	Yes
PALA9	Spermidine/putrescine transport system permease	Yes	No	Yes	No	Yes
WTJH36	Spermidine/putrescine ABC transporter	No	Yes	Yes	Yes	Yes
H05	Spermidine/putrescine ABC transporter	No	Yes	Yes	Yes	Yes
*Bacillus subtilis*						
SRCM102754	Putative ABC transporter ATP-binding protein, ABC transporter permease	No	Yes	No	No	Yes
PRO112	ABC transporter permease, ABC transporter ATP-binding protein	No	Yes	No	No	Yes
ms-2	No transport	No	No	No	No	No
SRCM103581	ABC transporter permease	No	Yes	No	No	No
29R7-12	Spermidine/putrescine ABC transporter	No	Yes	No	No	Yes
TR21	ABC transporter permease	No	Yes	No	No	Yes
ATC-3	ABC transporter permease	No	Yes	No	No	Yes
MB9_B4	ABC transporter permease	No	Yes	No	No	Yes
*Staphylococcus aureus*						
ATCC BAA-44	Putrescine export system ATP-binding protein	No	No	No	No	Yes
ATCC 6538	Putrescine export system ATP-binding protein	No	No	No	No	Yes
ATCC 9144	Putrescine export system ATP-binding protein	No	No	No	No	Yes
ATCC 12600	Putrescine export system ATP-binding protein	No	No	No	No	Yes
ATCC BAA-1680	Putrescine export system ATP-binding protein	No	No	No	No	Yes
ATCC 27660	Putrescine export system ATP-binding protein	No	No	No	No	Yes
ATCC25923	Putrescine export system ATP-binding protein	No	No	No	No	Yes
ATCC43300	Putrescine export system ATP-binding protein	No	No	No	No	Yes
KG-22	Spermidine/putrescine ABC transporter	No	No	No	No	No
JK3137	Spermidine/putrescine ABC transporter	No	No	No	No	No
MRSA—AMRF 5	Spermidine/putrescine ABC transporter	No	No	No	No	No
AR_0216	Spermidine/putrescine import ATP-binding protein	No	No	No	No	No
USA300-SUR16	Spermidine/putrescine ABC transporter	No	No	No	No	No
110900	Spermidine/putrescine ABC transporter	No	No	No	No	No
CMRSA-3	Spermidine/putrescine ABC transporter	No	No	No	No	No
*Escherichia coli*						
2010C-3347	No transport	No	No	No	No	No
W3110	Putrescine/proton symporter, putrescine transport protein	Yes	Yes	No	No	Yes
K-12 (MG1655)	Putrescine-ornithine antiporter, putrescine transporter, putative amine transport	Yes	Yes	No	No	Yes
117	Spermidine/putrescine ABC transporter, putrescine ABC transporter permease, putrescine-ornithine antiporter	Yes	Yes	No	No	Yes
DSM 103246	Spermidine/putrescine ABC transporter	Yes	Yes	No	No	Yes
CV261	Putrescine-ornithine antiporter, spermidine/putrescine ABC transporter, putrescine ABC transporter permease	Yes	Yes	No	No	Yes
STEC2018-553	Putrescine/proton symporter, putrescine/proton symporter, putrescine ABC transporter permease, putrescine-ornithine antiporter	Yes	Yes	No	No	Yes
2013C-3277	No transport	No	No	No	No	No
CFSAN027350	Putrescine-ornithine antiporter, putrescine/proton symporter, putrescine/proton symporter, spermidine/putrescine ABC transporter, putrescine ABC transporter permease	Yes	Yes	No	No	Yes
Ecol_AZ146	Putrescine-ornithine antiporter	Yes	Yes	No	No	Yes
2014C-3655	No transport	No	No	No	No	No
*Mycobacterium tuberculosis*						
SEA02010036P6C4	ABC transporter permease	No	No	No	No	No
1-0110P6c4	ABC transporter permease	No	No	No	No	No
MTB2	ABC transporter permease	No	No	No	No	No
H54	Spermidine/putrescine ABC transporter, ABC transporter permease	No	No	No	No	No
BLR-31d	ABC transporter permease, ABC transporter permease	No	No	No	No	No

## Data Availability

All data are included in this paper.
